# Host Immune Responses to a Viral Immune Modulating Protein: Immunogenicity of Viral Interleukin-10 in Rhesus Cytomegalovirus-Infected Rhesus Macaques

**DOI:** 10.1371/journal.pone.0037931

**Published:** 2012-05-24

**Authors:** Meghan K. Eberhardt, W. L. William Chang, Naomi J. Logsdon, Yujuan Yue, Mark R. Walter, Peter A. Barry

**Affiliations:** 1 Center for Comparative Medicine, University of California Davis, Davis, California, United States of America; 2 Department of Microbiology, University of Alabama at Birmingham, Birmingham, Alabama, United States of America; 3 Department of Pathology and Laboratory Medicine, University of California Davis, Davis, California, United States of America; 4 California National Primate Research Center, University of California Davis, Davis, California, United States of America; Emory University School of Medicine, United States of America

## Abstract

**Background:**

Considerable evidence has accumulated that multiple viruses, bacteria, and protozoa manipulate interleukin-10 (IL-10)-mediated signaling through the IL-10 receptor (IL-10R) in ways that could enable establishment of a persistent microbial infection. This suggests that inhibition of pathogen targeting of IL-10/IL-10R signaling could prevent microbial persistence. Human cytomegalovirus (HCMV) and rhesus cytomegalovirus (RhCMV) express a viral interleukin-10 (cmvIL-10 and rhcmvIL-10, respectively) with comparable immune modulating properties *in vitro* to that of their host's cellular IL-10 (cIL-10). A prior study noted that rhcmvIL-10 alters innate and adaptive immunity to RhCMV *in vivo*, consistent with a central role for rhcmvIL-10 during acute virus-host interactions. Since cmvIL-10 and rhcmvIL-10 are extremely divergent from the cIL-10 of their respective hosts, vaccine-mediated neutralization of their function could inhibit establishment of viral persistence without inhibition of cIL-10.

**Methods and Findings:**

As a prelude to evaluating cmvIL-10-based vaccines in humans, the rhesus macaque model of HCMV was used to interrogate peripheral and mucosal immune responses to rhcmvIL-10 in RhCMV-infected animals. ELISA were used to detect rhcmvIL-10-binding antibodies in plasma and saliva, and an IL-12-based bioassay was used to quantify plasma antibodies that neutralized rhcmvIL-10 function. rhcmvIL-10 is highly immunogenic during RhCMV infection, stimulating high avidity rhcmvIL-10-binding antibodies in the plasma of all infected animals. Most infected animals also exhibited plasma antibodies that partially neutralized rhcmvIL-10 function but did not cross-neutralize the function of rhesus cIL-10. Notably, minimally detectable rhcmvIL-10-binding antibodies were detected in saliva.

**Conclusion:**

This study demonstrates that rhcmvIL-10, as a surrogate for cmvIL-10, is a viable vaccine candidate because (1) it is highly immunogenic during natural RhCMV infection, and (2) neutralizing antibodies to rhcmvIL-10 do not cross-react with rhesus cIL-10. Exceedingly low rhcmvIL-10 antibodies in saliva further suggest that the oral mucosa, which is critical in RhCMV natural history, is associated with suboptimal anti-rhcmvIL-10 antibody responses.

## Introduction

Human cytomegalovirus is well recognized as a clinically relevant pathogen in those with immature or compromised immune systems, particularly congenitally infected fetus/newborns and immunosuppressed transplant recipients. Because of the pathogenic potential of HCMV in these and other populations, there have been four decades of calls to develop a vaccine that confers protective efficacy against HCMV infection and disease [1]–[5]. Primary vaccine research has focused on those HCMV proteins encoding epitopes for either neutralizing antibodies (glycoprotein B-gB) or strong cellular immune responses (phosphoprotein 65 and immediate early-1), based on humoral and cellular immune correlates of protection against congenital infection and transplantation-associated HCMV disease, respectively [6], [7]. Two vaccine trials based on recombinant gB vaccination in seronegative pregnant women and seronegative renal transplant recipients demonstrated partial, but incomplete, protection. One possibility for boosting protective efficacy beyond that observed in the gB trials is to include additional HCMV antigens to broadly target vulnerable components of viral replication. Additionally, an important related issue is whether there is a need to direct the vaccine immune responses to mucosal sites to maximize the immediacy of antiviral responses that prevent dissemination of progeny virions beyond the site of challenge virus infection. Our current study in the rhesus macaque model of HCMV infection was initiated to begin to address these two issues of HCMV vaccine design based on the following considerations.

HCMV immune modulating proteins that disrupt host cell functions, including antigen presentation, signaling, trafficking, activation, and metabolism, have not been evaluated as vaccine candidates. A large percentage of the coding capacity of the HCMV genome is devoted to these proteins, and *in vitro* studies strongly suggest that HCMV immune modulating proteins collectively could skew host immunity during critical junctures of HCMV infection *in vivo*. Limited studies have observed cellular immune responses specific to presumptive HCMV immune modulating proteins, including those that disrupt MHC class I antigen presentation (US2, US3, US6, US11), and those that inhibit NK function (UL16, UL18) [8], [9]. Antibody responses have also been detected against UL111A (cmvIL-10) in some HCMV-infected individuals [10]. One group profiling HCMV T cell responses noted that, based on the *in vitro* phenotype of the immune modulating proteins and their potential role *in vivo*, “Future investigations will need to assess the levels of protection afforded by responses directed toward these epitopes” [8].

Mucosal surfaces are the sites of both primary infection and reinfection. Analytic modeling of published congenital transmission rates has led to the conclusion that ∼75% of congenital infections in the United States occur in women with preconceptional immunity to HCMV [11]. The study did not determine whether maternal-to-fetal transmission was the result of maternal reactivation of preexisting virus or reinfection with a horizontally transmitted virus. Based on multiple studies demonstrating reinfection of seroimmune pregnant women, including in populations with universal seroprevalence, it is clear that prior immunity is only partially protective against reinfection [12]–[19]. A salient point of these studies is that HCMV reinfection in these instances was the result of mucosal exposure to horizontally acquired virus, implying that mucosal antiviral immune responses were insufficient to contain viral reinfection locally.

With the long-term goal of testing *in vivo* the concept of immune modulating proteins as vaccine candidates, immune responses to rhcmvIL-10 (RhUL111A) were evaluated in healthy RhCMV-infected rhesus macaques persistently infected with wild-type RhCMV. Studies have shown that rhcmvIL-10 plays a dynamic role in viral immune modulation, mimicking cellular IL-10 functions *in vitro* and altering innate and adaptive immune responses to viral antigens *in vivo* [20]. Microbial manipulation of IL-10 appears to be a common feature of many persistent viruses (*e.g.*, murine CMV, LCMV, hepatitis B and C viruses), bacteria (*e.g.*, *Chlamydia trachomatis, and Listeria monocytogenes*), and protozoa (*e.g.*, Leishmania and *Plasmodium* species) [21]–[36], and commensal bacteria [37]–[39]. Thus, there is extensive precedent to focus on rhcmvIL-10 and cmvIL-10 as central players in primate CMV natural history. The potential viability of using rhcmvIL-10 in a vaccine was recently described for rhcmvIL-10 [40]. Structural biology was used to engineer biologically inactive mutants of rhcmvIL-10 that do not bind to the IL-10 high-affinity receptor and, therefore, lack wild-type functional activity. To provide a foundation for evaluating the immunogenicity of non-functional versions of rhcmvIL-10 in RhCMV-uninfected animals, peripheral and mucosal immune responses to wild-type rhcmvIL-10 were surveyed in RhCMV-infected juvenile and adult rhesus macaques.

## Results

### rhcmvIL-10-binding antibodies in RhCMV-infected monkeys

**Figure 1:**
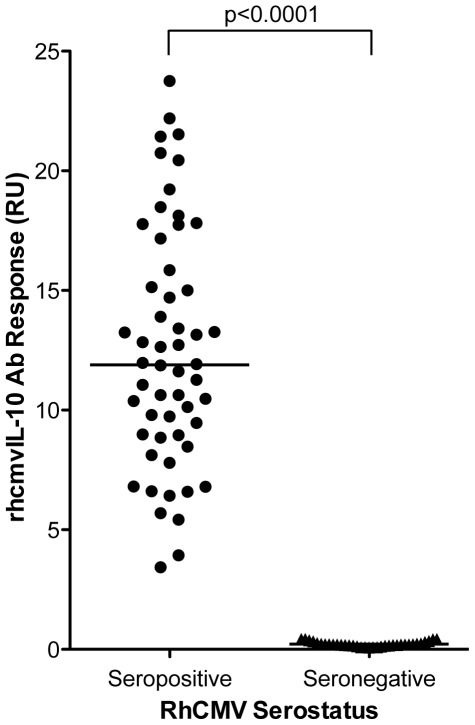
rhcmvIL-10 antibody seroprevalence in rhesus macaques. Plasma samples from macaques confirmed positive or negative for RhCMV (54 RhCMV seropositive, 35 seronegative) were screened for the presence of rhcmvIL-10 antibodies by rhcmvIL-10 ELISA. RhCMV seropositive samples had significantly higher rhcmvIL-10 antibody levels than seronegative samples (p<0.0001; two-tailed unpaired student's t-test) with a range of 3 to 24 Relative Units (RU). The line represents the median for the group.

**Figure 2:**
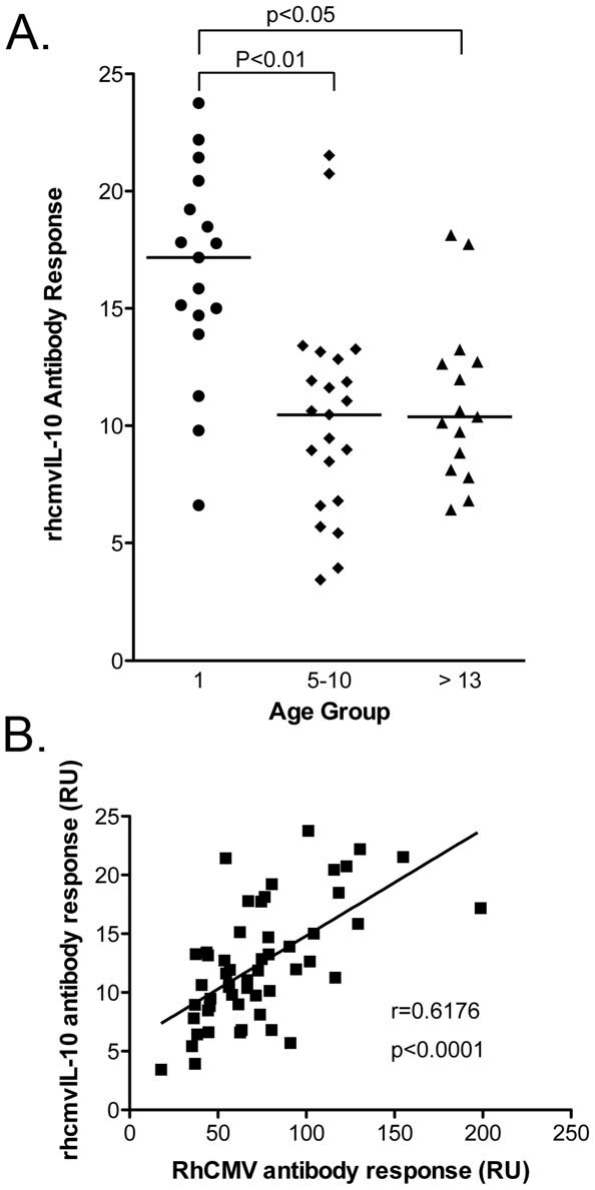
rhcmvIL-10 antibody response. (A) An age comparison of rhcmvIL-10 responses in 54 macaques seropositive for RhCMV stratified into 3 age groups; infant (≤1 year), adult (5–10 years) and aged (≥13 years). The infants had significantly higher antibody titers than the adults and aged (p<0.01, p<0.05 respectively). Statistical analysis between age groups was done using Kruskal-Wallis test with Dunn's Multiple Comparison. There was no significant difference between the adult and aged animal groups. (B) Linear regression analysis of RhCMV antibodies and rhcmvIL-10 antibodies from 53 rhesus macaques reported in relative units (RU). There was a significant correlation between total RhCMV titers and rhcmvIL-10 antibody titers (Pearson r = 0.6176, p<0.0001) with RhCMV antibody titers being significantly predictive of rhcmvIL-10 antibody production.

A rhcmvIL-10 ELISA was developed to characterize the kinetics and magnitude of rhcmvIL-10-specific binding antibodies in macaques naturally exposed to RhCMV circulating in outdoor-housed cohorts (see Materials and Methods for details). Plasma samples from outdoor-housed rhesus macaques, which were confirmed to be either RhCMV seropositive (N = 54) or seronegative (N = 35) by an ELISA using RhCMV-infected cell extract as antigen were randomly chosen and screened by ELISA for the presence of rhcmvIL-10 binding antibodies. All RhCMV antibody-positive macaques were positive for rhcmvIL-10-binding antibodies, while all RhCMV antibody-negative samples were also negative for rhcmvIL-10 antibodies (p<0.0001) (Fig. 1). rhcmvIL-10-binding antibody titers in the RhCMV antibody-positive population ranged from 3–24 relative units (RU) with a median of 11.9 RU. When rhcmvIL-10 antibody titers were stratified by the age of the animal, (≤1, 5–10, and >13 years, corresponding to infant (N = 17), adult (N = 22), and aged (N = 15) animals, respectively), significantly higher rhcmvIL-10-specific titers were detected in the infants, compared to the adult and aged groups (p<0.001, p<0.01 respectively) (Fig. 2A). The rhcmvIL-10 titers in the adult and aged animals were indistinguishable. Previous seroepidemiological studies have demonstrated that there is 50% seroconversion to RhCMV infection by 6 months of age and complete seroconversion around 1 year in outdoor, group-housed macaques, similar to those included in this study [41]. Thus, the adult and aged animals had, most probably, been infected long-term (>4–>12 years) with RhCMV. The relative increased antibody responses to rhcmvIL-10 in the infants did not appear to be specific to this particular viral protein. A similar age-related pattern of seroreactivity was observed when an antigen preparation, consisting of a total protein lysate of RhCMV-infected cells, was used instead (data not shown). There was a strong correlation between rhcmvIL-10 titers and RhCMV antibody titers (Pearson, r = 0.6176, p<0.0001) (Fig. 2B), indicating that the magnitude of rhcmvIL-10 antibody titers reflected the magnitude of antibody titers to total RhCMV antigens.

### Avidity of rhcmvIL-10 antibodies

**Figure 3:**
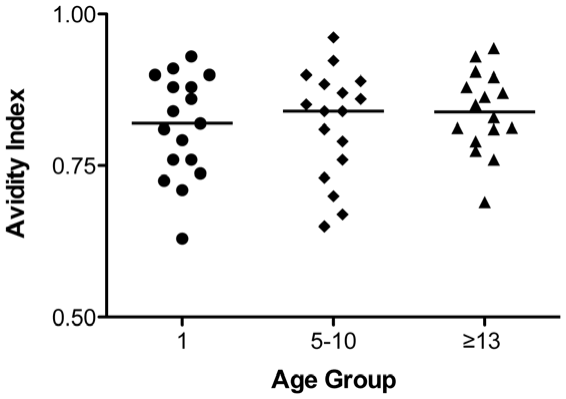
rhcmvIL-10 antibody avidity. Plasma samples from RhCMV-infected animals ≤1 (N = 17), 5–10 (N = 17), and ≥13 (N = 16) years of age were assayed for avidity of rhcmvIL-10 antibodies (see Materials and Methods). The average avidity ratio for each group was 0.82 (≤1 year), 0.84 (5–10 years), and 0.84 (≥13 years). There was no significant difference between age groups.

The binding strength of antibodies was evaluated for 50 RhCMV-positive macaques using an ELISA avidity assay with a 6 M Urea wash. All RhCMV-infected animals exhibited high avidity indices to rhcmvIL-10, ranging from 0.63 to 0.96 with an average of 0.83 (standard deviation = 0.076) (Fig. 3). These results were consistent with what has previously been found in overall RhCMV antibody avidity [42]. No differences in avidity were detected between the age groups.

### rhcmvIL-10-neutralizing antibody titers

**Figure 4:**
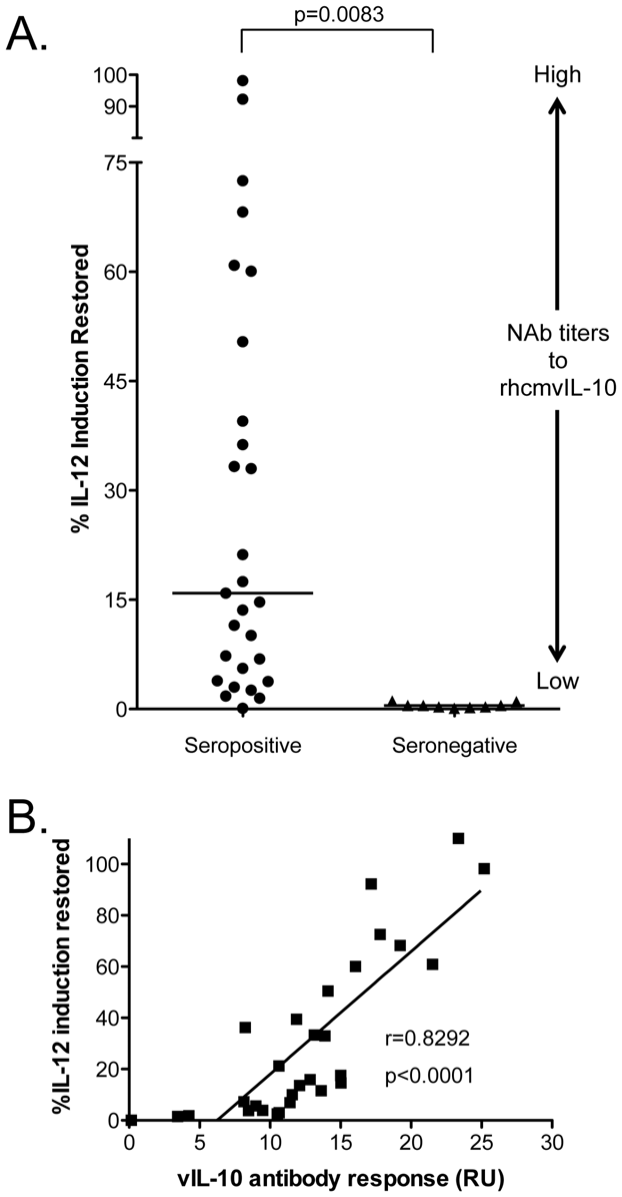
rhcmvIL-10 antibodies neutralized rhcmvIL-10 activity in stimulated PBMC. rhcmvIL-10 neutralization was determined by incubating LPS-stimulated PBMC with either rhcmvIL-10 and RhCMV seropositive plasma, or rhcmvIL-10 only. The % difference of PBMC IL-12 secretions in the rhcmvIL-10/ plasma versus plasma only (measured by ELISA) was termed “% IL-12 induction restored”. (A) Seropositive plasma sample neutralization of rhcmvIL-10 resulted in a range of 0–100% IL-12 induction restored with a significant difference between the RhCMV seropositive and seronegative samples (p = 0.0083; two-tailed student's t-test). The relative relation between %IL-12 restored and strength of NAB is shown on the right. (B) % IL-12 induction restored was found to be directly correlated to rhcmvIL-10 antibody titers (measured in RU) in rhesus macaque seropositive plasma (Pearson r = 0.8292. p<0.0001).

rhcmvIL-10 antibody responses in plasma were quantified by an *in vitro* assay to determine if rhcmvIL-10-binding antibodies also neutralized its functional activity. Plasma samples from RhCMV-immune animals were evaluated for the ability to neutralize rhcmvIL-10-mediated responses in activated peripheral blood mononuclear cells (PBMC). In brief, the assay compared the level of IL-12 synthesized by lipopolysaccharide (LPS)-activated PBMC following incubation with either rhcmvIL-10 diluted in rhesus plasma or plasma alone (Fig. 4). Preliminary assays verified that LPS-stimulated PBMC secreted high amounts of IL-12 (an average of 1.5 ng/2×10^5^ cells), which was abrogated when the cells were pre-treated with rhcmvIL-10 (data not shown). Since rhcmvIL-10 inhibits the production of IL-12 in LPS-treated PBMC, antibody-mediated neutralization of rhcmvIL-10 activity was measured by increased levels of IL-12 production following incubation of LPS-activated PBMC with rhcmvIL-10. If a plasma sample lacked rhcmvIL-10 neutralizing antibodies, IL-12 expression would not be restored. Based on these results, IL-12 induction was used to quantify the ability of rhcmvIL-10 antibodies to bind to and neutralize rhcmvIL-10 activity. Plasma samples from 26 seropositive and 9 seronegative rhesus macaques were selected from the pool of those previously assayed by the rhcmvIL-10-binding ELISA and assessed for the ability to neutralize rhcmvIL-10 activity (Fig. 4). RhCMV seropositive animals exhibited a wide range of neutralizing activity (0–100% IL-12 induction restored) with a median at 15.9% IL-12 induction restored (Fig. 4A). No neutralization of rhcmvIL-10 was detected using plasma from RhCMV-uninfected monkeys. rhcmvIL-10 neutralizing titers exhibited a positive correlation with rhcmvIL-10 antibody titers (Fig. 4B; r = 0.8292, p<0.0001). Infants showed slightly higher rhcmvIL-10 neutralizing titers than adults (data not shown), consistent with the higher rhcmvIL-10 antibody titers observed in this age group (Fig. 2B).

### Mucosal Antibody Levels

**Figure 5:**
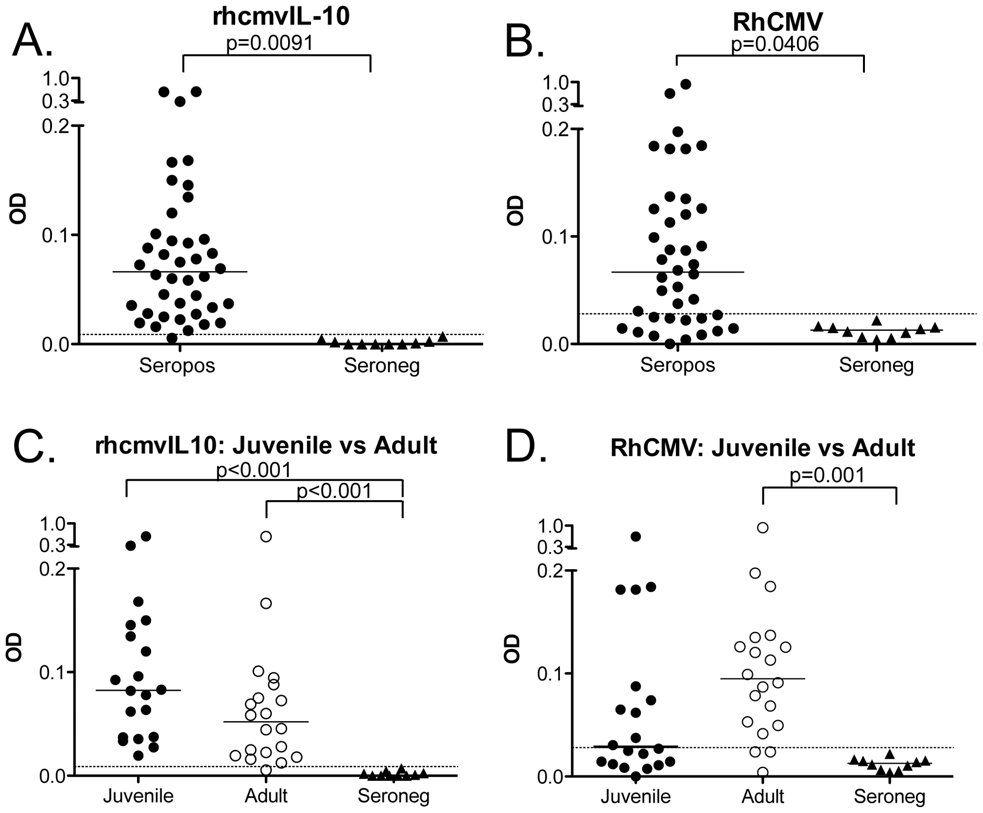
Analysis of mucosal IgG. Saliva samples from RhCMV seropositive and seronegative animals were analyzed by ELISA for IgG responses to rhcmvIL-10 (A) and RhCMV (B) (one-tailed student's t-test). RhCMV seropositive animals were stratified by age as either juvenile (2–4 years) or aged (≥13 years) for IgG responses to rhcmvIL-10 (C) and RhCMV (D), in comparison to responses in RhCMV seronegative animals. The dashed line is the cut-off for samples to be considered positive. Statistical analysis between age groups was done using Kruskal-Wallis test with Dunn's Multiple Comparison.

**Figure 6:**
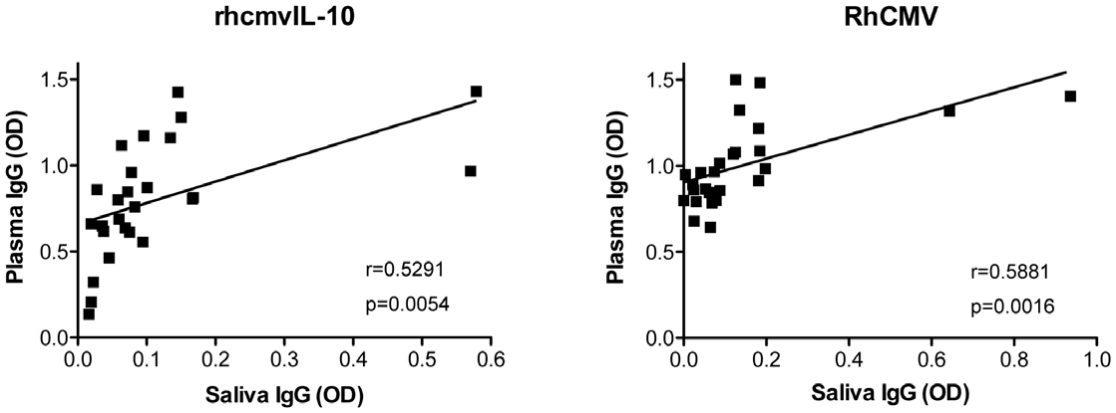
Correlation of saliva IgG and plasma IgG responses to rhcmvIL-10 and RhCMV. The optical density (OD) of the saliva and plasma IgG responses to rhcmvIL-10 (left) and RhCMV antigens (right) are presented.

Since HCMV infection and shedding occur at mucosal surfaces, local immune responses in the mucosa are likely to be important for mediating protection against reinfection and in determining the frequency and magnitude of shedding. Saliva samples from 20 RhCMV seropositive juveniles (2–4 years), 20 RhCMV seropositive adults (>10 years old), and 10 RhCMV seronegative macaques were analyzed by ELISA for IgG responses specific to RhCMV and rhcmvIL-10. Saliva IgG was evaluated to provide a surrogate measure of tissue levels of rhcmvIL-10 NAb either secreted by plasma cells in the submucosa or transudated from plasma. IgA levels were not analyzed since both rhcmvIL-10 and cmvIL-10 are secreted proteins [20], [43]. In addition, there is no evidence that cmvIL-10 is present in the HCMV virion [44]. Saliva antibody titers to rhcmvIL-10 and RhCMV were exceedingly low in the RhCMV seropositive animals (Figs. 5A and 5B), yet a threshold for determining a positive response was established from the responses in the seronegative animals. Samples were considered positive if the optical density (OD) equaled or exceeded the mean OD of the seronegative animals plus 3 standard deviations of the mean. Small differences were noted between the juvenile and adult responses in saliva (Figs. 5C and 5D). Whereas rhcmvIL-10 responses were slightly higher in juveniles and RhCMV-specific responses were higher in adults, the differences were not statistically significant. Notably, only 50% of the juveniles had detectable RhCMV antibodies in saliva, compared with 85% of adults with detectable antibodies in saliva. Based on the dilutions used for the saliva and plasma, and the ELISA responses, antibody levels in saliva were ∼0.025% of those found in their corresponding plasma samples (Fig. 5 and data not shown). Studies in humans indicate that saliva IgG levels are ∼0.1% of IgG titers in plasma [45]–[47]. The IgG responses in saliva were directly correlated with those in plasma for both rhcmvIL-10 (r = 0.5291, p = 0.0054) and RhCMV antigens (r = 0.5881, p = 0.0016) (Fig. 6). These results were consistent with previous studies demonstrating that mucosal IgG is primarily derived from transudated plasma IgG [48]–[50].

### Absence of cross-reactive neutralization to rhesus cIL-10

**Figure 7:**
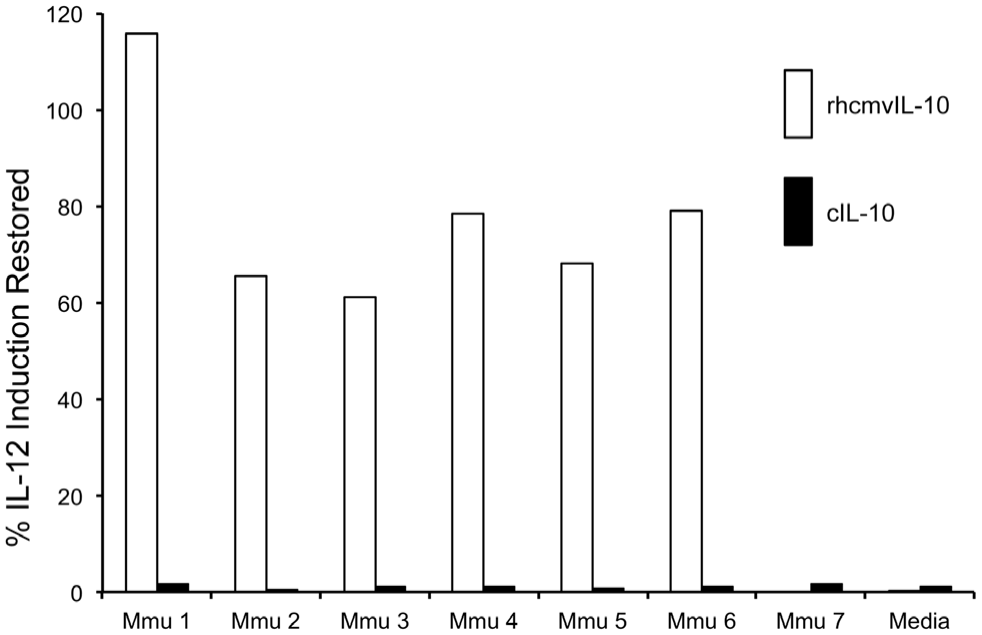
Absence of cross-reactivity of rhcmvIL-10 neutralizing antibodies to rhesus cellular IL-10 (cIL-10). Plasma from six RhCMV-infected (Mmu 1–Mmu 6) monkeys and one RhCMV-uninfected monkey (Mmu 7) were assayed for the ability to neutralize functional activity of rhcmvIL-10 (open column) and cIL-10 (filled column). Plasma from Mmu 1–Mmu 6 neutralized rhcmvIL-10 functionality but had no neutralization of cIL-10. Media: no plasma was added to the reaction.

Considering the extensive sequence divergence between rhcmvIL-10 and rhesus cIL-10 (25% amino acid identity), there is little likelihood that antibodies specific to rhcmvIL-10 would cross-react with epitopes (linear or conformational) within cIL-10. Further, since RhCMV infection is subclinical in rhesus macaques, there is no clinical evidence consistent with development of autoimmune antibodies to rhesus cIL-10 coincident with development of antibodies to rhcmvIL-10. To rule out the hypothetical possibility of cross-reactivity, such as those directed at potential conformational epitopes, rhcmvIL-10 neutralization assays were performed to determine whether plasma samples that neutralized rhcmvIL-10 similarly neutralized the immunosuppressive activity of rhesus cIL-10 on LPS-activated PBMC. Plasma from six RhCMV-infected macaques that neutralized rhcmvIL-10 activity from 60–100% had undetectable neutralizing activity against rhesus cIL-10 (Fig. 7). IL-12 remained undetectable in the supernatant of LPS-activated PBMC when co-incubated with cIL-10 and plasma from these same animals. These results strongly indicate that antibodies specific to rhcmvIL-10 generated during the course of RhCMV infection do not cross-react with endogenous cIL-10.

### rhcmIL-10-specific T-cell responses

To evaluate the prevalence and magnitude of the cellular response to rhcmvIL-10, FLOW cytometry was used to determine the frequency and magnitude of rhcmvIL-10-specific T-cell responses. PBMC from 23 out-door housed, RhCMV-infected animals were stimulated with either total RhCMV antigen or rhcmvIL-10. For these assays, two non-functional versions of rhcmvIL-10 (termed M1 and M2) [40] were used to avoid the immunosuppressive effects of wild-type rhcmvIL-10. Only 22% (N = 5 animals) of RhCMV-infected animals had detectable rhcmvIL-10-specific T cell responses (Table 1). 13% (N = 3) exhibited a CD4^+^/IFN-γ^+^ T-cell response, and 8% (N = 2) had a CD8^+^ T-cell response (IFN-γ). As a confirmation that the population had RhCMV-specific cellular responses, PBMC were also stimulated with heat-inactivated RhCMV virion. 96% of animals (N = 22) had a RhCMV-specific CD4^+^/IFN-γ^+^ T-cell response while 30% had a CD8^+^/IFN-γ^+^. In sum, while rhcmvIL-10 stimulates antibody responses in all RhCMV-infected animals, only a minority of infected animals developed detectable cellular responses.

**Table 1:** IFNγ responses in CD4^+^ and CD8^+^ T cells from 24 RhCMV-infected macaques following coincubation with either RhCMV or rhcmvIL-10 M1/M2 antigens.

	CD4		CD8	
Antigen	% of animals with IFNγ^+^ CD4^+^ T cells (#)	Mean Percentage of IFNγ^+^ CD4^+^ T cells (SD)	% of animals with IFNγ^+^ CD8^+^ T cells (#)	Mean Percentage of IFNγ^+^ CD8^+^ T cells (SD)
RhCMV	96 (22)	0.29 (0.15)	30 (7)	0.06 (0.04)
rhcmvIL-10M1/M2	13 (3)	0.04 (0.017)	8 (2)	0.015 (0.002)

## Discussion

Multiple studies have documented that HCMV can reinfect immune competent individuals with prior HCMV infection [11]–[19], [51], [52]. One study demonstrated that 30% of seropositive women that were longitudinally evaluated developed novel HCMV antibody specificities, equivalent to an annual rate of HCMV reinfection of 10% [53]. This is particularly remarkable because ∼10% of memory T cells in healthy long-term HCMV carriers are HCMV-specific, and neutralizing antibodies are generated against multiple viral glycoproteins [9], [54]–[58]. Taken together, studies of reinfection indicate a viral mechanism to surmount preexisting immune memory to initiate a new infection, which implies that infection of an immune host may be independent of whether antiviral immunity is from prior infection or prior vaccination.

Accordingly, one strategy to increase vaccine-mediated protective efficacy beyond that afforded by gB alone would be to target the HCMV mechanisms responsible for immune evasion, particularly those that are operative during the initial virus-host interactions. Multiple *in vitro* and *in vivo* studies of cmvIL-10 and rhcmvIL-10 support a model in which the viral ortholog of cIL-10, expressed at the site of infection, subverts local and systemic immunity to enable dissemination of progeny virions to distal sites [20], [59]–[66]. This study demonstrates that rhcmvIL-10 is highly immunogenic during natural RhCMV infection, and infection-generated antibodies that neutralize rhcmvIL-10 function are specific to the viral IL-10 ortholog; there is no evidence that there is any cross-reactivity with rhesus cIL-10. By extension, rhcmvIL-10 vaccine-induced neutralizing antibodies in a naïve host should not cross react with cIL-10. Since immunization with a functional version of rhcmvIL-10 could skew immune responses because of the immunosuppressive properties of rhcmvIL-10, an alternative vaccine strategy has recently been described [67]. Two mutated versions of rhcmvIL-10 (M1 and M2) were generated, each containing two point mutations within the IL-10R binding site. Neither mutated version of rhcmvIL-10 bound soluble IL-10R, and neither had functional activity on activated PBMC. In addition, immunization of RhCMV-infected monkeys with M1 and M2 boosted preexisting rhcmvIL-10-binding and neutralizing antibodies. This proposed vaccine approach for rhcmvIL-10, and by extension cmvIL-10, takes advantage of the extreme sequence divergence between rhcmvIL-10 and cmvIL-10 from the cIL-10 of their macaque and human host, respectively [68]. While primate cIL-10 proteins share >95% identity, the viral IL-10 orthologs are nearly as divergent from each other (31% identity) as they are from the cIL-10 of their host (25–27%). This kind of approach is not likely to work for the Epstein-Barr Virus IL-10 protein (ebvIL-10; Accession # P03180), which retains 92% identity with human cIL-10 (P22301). Antibodies that bind human cIL-10 also bind ebvL-10, and anti-ebvIL-10 antibodies bind cIL-10 [69]. This same study also demonstrated that monoclonal antibodies to cmvIL-10 do not bind human cIL-10 or ebvIL-10. HCMV also expresses a Latency Associated cmvIL-10 (LAcmvIL-10) that is the result of translation of a partially spliced transcript of cmvIL-10 [70]. It is not known at this time whether a comparable LArhcmvIL-10 is expressed in RhCMV-infected cells. Since evidence suggests that LAcmvIL-10 does not bind to IL-10R1 [71], it is probable that antibodies that neutralize cmvIL-10 engagement of IL-10R1 would likely not neutralize LAcmvIL-10, although this remains to be verified.

The characterization of rhcmvIL-10 immune responses in RhCMV-infected macaques provides two important benchmarks for vaccination of naïve macaques with non-functional versions of rhcmvIL-10: generation of (1) high titers of plasma antibodies that neutralize rhcmvIL-10 function, and (2) more robust mucosal anti-rhcmvIL-10 responses. This latter point is a reflection of the importance of mucosal surfaces in HCMV and RhCMV natural history. Since the overall rate of congenital infection is ∼0.6% [72], the overwhelming preponderance of primary infections results from horizontal transmission of HCMV in bodily fluids of an infected person to an epithelial cell surface, such as the oral and genital mucosa, of an uninfected recipient. These also serve as portals for reinfection of immune hosts, and as sources of viral shedding in long-term infected individuals. An ideal vaccine would absolutely contain virus locally after mucosal exposure and block dissemination to such distal sites as the maternal-fetal interface. The absence of complete protection in the gB vaccine trials and the ability of HCMV to reinfect immune hosts implicate a failure of local immunity to contain “challenge” virus. Consequently, boosting mucosal immune responses could increase the protective efficacy of vaccine-generated immunity. Since antibody responses that neutralize rhcmvIL-10 function are linearly related to the binding antibody responses, the minimally detectable rhcmvIL-10-binding antibodies in saliva imply that local antibody responses at the oral mucosa would be insufficient to neutralize soluble rhcmvIL-10 after exposure to a reinfecting virus. Moreover, the direct correlation between IgG responses in plasma and saliva suggests that maximizing plasma titers of antibodies that neutralize rhcmvIL-10 could be reflected by a commensurate increase of such antibodies in mucosal fluids, perhaps increasing protection against horizontally transmitted virus. The results herein establish a framework to measure whether vaccination with non-functional rhcmvIL-10 stimulates greater level of rhcmvIL-10-specific antibody responses above that provided by prior infection with RhCMV.

## Materials and Methods

### Ethics statement

The University of California, Davis (UC Davis) is accredited by the Association for Assessment and Accreditation of Laboratory Animals Care (AAALAC, Animal Assurance #: A3433-01), a private, nonprofit group that promotes the humane treatment of animals in science through voluntary accreditation. UC Davis is one of more than 640 research institutions and other organizations that have earned AAALAC accreditation, demonstrating its commitment to responsible animal care and use. In addition, the CNPRC receives unannounced inspections by the U.S. Department of Agriculture, as required by the Animal Welfare Act, and inspections by the Food and Drug Administration. This study was carried out in strict accordance with the recommendations in the Guide for the Care and Use of Laboratory Animals of the National Institutes of Health and in accordance with the recommendations of the Weatherall report, “The use of nonhuman primates in research”. The Institutional Animal Care and Use Committee of UC Davis approved in advance all animal use protocols. Multiple veterinarians and animal care technicians provided state-of-the-art care and research support for these studies. The animals were monitored by veterinarians and trained animal care staff every day and during all procedures. Animals were housed in outdoor corrals (∼2,025 square meters) at an average density of 145 macaques per corral with ages of the animals ranging from neonate to aged adult (>14 years). Access to water was freely available 24 hours per day, and fresh food was provided in feeding bins twice each day. Each corral contained protection from wind, sun, and rain that was sufficient to protect every animal. Thermostat-controlled heaters were triggered when the temperature dropped to 2°C during winter, and thermostat-controlled, overhead water misters were triggered when the temperature reached 32°C. Electrical systems were protected by back-up diesel generators in the advent of a systemic power outage. Animals were anesthetized with ketamine during blood and saliva collection to prevent any suffering. Care was taken to ensure that the animals were adequately sedated under all conditions, as assessed by the veterinarian and/or animal care staff. The specific animal use protocol for this study was 15137.

### Animals

Healthy, genetically outbred rhesus macaques (*Macaca mulatta*) from the California National Primate Research Center, confirmed to be either RhCMV seronegative or RhCMV seropositive, were used for these studies. Oral swabs were collected according to our published procedures [73]. The Institutional Animal Care and Use Committee of the University of California, Davis approved all animal protocols in advance of any procedures.

### Expression and purification of rhcmvIL-10

Purification of wild-type rhcmvIL-10 and the non-functional versions (M1 and M2) for use in ELISA and *in vitro* assays has been previously described [40].

### rhcmvIL-10 ELISA

Antibodies against rhcmvIL-10 were characterized by ELISA by modifying a previously published protocol [74]. Pilot assays were performed to optimize the amount of coating antigen and secondary antibody concentration necessary to give a broad linear range of reactivity and to maximize the distinction between plasma samples from RhCMV-infected and uninfected animals. Based on these assays (data not shown), 96-well microplates (Immulon 4 HBX, Dynex Technologies Inc.) were coated overnight at 4°C with nickel affinity-purified rhcmvIL-10 (12.5 ng/well) in phosphate buffered saline (PBS) (Sigma)/0.375% sodium bicarbonate (GIBCO). Each plate was subsequently washed 6 times with PBS/0.05% Tween 20 (Sigma) (PBS-T) and blocked with 300 µl/well PBS/1% bovine serum albumin (BSA) (Sigma) for 2 hours at 25°C in a temperature-controlled incubator. After washing the plates 6 times with PBS-T, 100 µl of a 1∶100 dilution of rhesus monkeys plasma (in PBS-T/1% BSA), or 100 µl of oral swab in PBS (1∶10 final dilution) was added to each well and incubated at 25°C for 2 hours. Plasma samples were from rhesus macaques serologically confirmed to be infected or uninfected with RhCMV. Each sample was assayed in duplicate. The plates were subsequently washed 6 times with PBS-T wash buffer and loaded with 100 µl/well of a 1∶60,000 dilution of peroxidase-conjugated goat-anti-monkey IgG (Kirkegaard & Perry Laboratories, Inc - KPL) and incubated at 25°C for 1 hour. The plates were then washed 6 times with PBS-T wash buffer and 100 µl/well of tetramethylbenzidine liquid substrate (TMB) (Sigma) was added and incubated for 30 min at 25°C. TMB color development was stopped by the addition of 50 µl/well of 0.5 M sulfuric acid. After a 5-minute incubation at room temperature, color development was quantified spectrophotometrically at a wavelength of 450 nm on a Model 680 microplate reader (BioRad). Relative units (RU) were quantified using a standard curve of 10-fold serial dilutions of plasma from a rhesus macaque immunized with rhcmvIL-10 [40]. The threshold for a sample to be considered positive for a specific rhcmvIL-10 antibody response (RU = 1) was set at 6 standard deviations above the control seronegative mean optical density derived from 30 seronegative samples.

### Avidity assay of antibodies to rhcmvIL-10

Avidity binding of rhcmvIL-10 antibodies was assayed similarly to the ELISA protocol, except that after the primary 2-hour incubation with diluted plasma, the wells were incubated in freshly prepared 6 M urea for five minutes at room temperature, and then washed extensively with PBS-T. Secondary goat anti-monkey antibody was then added for 1 hour, and the plates were washed and processed for colorimetric development according to the ELISA protocol. The Avidity Index (AI) was calculated by dividing the mean optical density of a sample treated with 6 M urea by the mean optical density of the sample not treated with 6 M urea.

### Neutralization of rhcmvIL-10 function in vitro

Plasma samples from RhCMV seropositive and seronegative macaques were diluted (1∶4,000) in RPMI/10% fetal bovine serum (600 µL final volume) in the presence or absence of recombinant rhcmvIL-10 (0.5 ng/mL) for 3 hours at 37°C. This dilution of plasma was necessary due to the presence in plasma of endogenous inhibitory factors in less dilute plasma that inhibited IL-12 production by activated PBMC. 200 µL of the plasma +/− rhcmvIL-10 mixtures were then incubated (each in duplicate) with 4×10^5^ Ficoll-purified PBMC/well in a 96 well U-bottom plate (Falcon) for 30 minutes in a humidified 37°C incubator (5% CO_2_). LPS (from *E. coli* O127:B8; Sigma) was then added to the cells (5 µg/mL final concentration), and the cells were then incubated 24 hours at 37°C (5% CO_2_). The supernatant was collected the following day and stored at −80°C until assayed for IL-12 production. Rhesus IL-12 secretion by LPS-activated PBMC was measured by ELISA (U-Cytech, Netherlands), according to the manufacturer's protocol with slight variations. Briefly, 96-well microplates were coated with the supplied IL-12 antibody pair (p40+p70) and incubated overnight at 4°C. The plates were then washed 6× with PBS-T and incubated with PBS/1% BSA blocking buffer for 60 minutes at 37°C. The buffer was removed, 100 µL/well of PBMC supernatant was added, and the cell mixture was incubated at 4° overnight. The plates were then washed 6 times with PBS-T wash buffer, 100 µL/well of anti-monkey ELISA detector antibody was added, and the cells were incubated 1 hr at 37°C. After washing, 100 µL/well of streptavidin-HR polymer (SPP) conjugate (U-Cytech) was added and incubated at 37°C for 1 hr. After washing, TMB substrate (100 µL/well) was added, and the plates were incubated at 25°C for 11 min. Color development was stopped by the addition of 0.5 M sulfuric acid (50 µL/well). Following a 5-minute incubation (25°C), the plates were read at a wavelength of 450 nm on a Model 680 microplate reader (BioRad). Concentrations of IL-12 were quantified using a 2-fold serially diluted recombinant IL-12 standard (U-Cytech) that was included on each plate. Neutralization was calculated as the inverse of the ratio of (IL-12 concentration+rhcmvIL-10+plasma)/(IL-12 concentration+plasma only) and was expressed as the “percent (%) IL-12 induction restored”.

### PBMC immunostaining

To evaluate T-cell responses to rhcmvIL-10, cryogenially preserved PBMC were thawed and rested overnight at 37° in complete RPMI medium containing 10% endotoxin-free FCS, 2 mM L-glutamine, 100 U/mL penicillin, 100 µg/mL streptomycin, 50 µM 2-mercaptoethanol, and 10 mM Hepes and stained following our published protocol [20] with slight modifications. Briefly, PBMC (2.5×10^6^ cells/mL in 48-well plates) were treated with either heat-inactivated RhCMV virion (10.0 µg/mL), non-functional rhcmvIL-10 proteins rhcmvIL-10M1 and rhcmvIL-10M2 (5.0 µg/mL each), or media only in the presence of co-stimulatory monoclonal antibodies to CD28 (clone 28.2) and CD49d (clone 9F10) (5 µg/mL each; ebiosciences) for 6.5 hours with the addition of Golgistop and Golgiplug after the first 1.5 hours. Surface staining was done using directly conjugated monoclonal antibodies against human (rhesus-macaques cross-reactive) CD3 (clone SP34-2), CD4 (clone L200) and CD8 (Clone SK1) (BD Biosciences). Cells were then fixed, permeabalized using the Fixation/Permeabilization Kit (BD Biosciences) and stained internally for IFN-γ (clone 4S.B3; BD Biosciences). A background baseline value was established for each animal by running a parallel sample without antigen stimulation. This value was the subtracted from the corresponding antigen stimulated sample.

### Flow Cytometry

Four-color flow cytometry was performed using the FACSCalibur with CellQuest software (BD Biosciences). Results were analyzed and displayed using FlowJo software (Tree Star).

### Statistics

All ELISA based OD results were converted into IgG relative units using a log-log regression model equation. The statistical analysis program Prism 4 was used for all statistical analyses. All corral surveys for the presence of RhCMV and rhcmvIL-10 antibodies were analyzed using the student's t-test (∝ = 0.05). All significant correlations were determined using Pearson's correlation coefficient analysis. One-way ANOVA and Kruskal-Wallis test with Dunn's Multiple Comparison (∝ = 0.05) were used for all age-matched analyses.
